# Optimal strategies to consider when peer reviewing a systematic review and meta-analysis

**DOI:** 10.1186/s12916-015-0509-y

**Published:** 2015-11-02

**Authors:** David Moher

**Affiliations:** Clinical Epidemiology Program, Ottawa Hospital Research Institute, General Campus, 501 Smyth Rd, Room L1288, Ottawa, ON K1H 8L6 Canada; School of Epidemiology, Public Health and Preventive Medicine, University of Ottawa, Ottawa, Canada; Canadian EQUATOR Centre, Ottawa, Canada

**Keywords:** Meta-analysis, Peer review, PRISMA, Reporting guidelines, Systematic review, The EQUATOR Network

## Abstract

Systematic reviews are popular. A recent estimate indicates that 11 new systematic reviews are published daily. Nevertheless, evidence indicates that the quality of reporting of systematic reviews is not optimal. One likely reason is that the authors’ reports have received inadequate peer review. There are now many different types of systematic reviews and peer reviewing them can be enhanced by using a reporting guideline to supplement whatever template the journal editors have asked you, as a peer reviewer, to use. Additionally, keeping up with the current literature, whether as a content expert or being aware of advances in systematic review methods is likely be make for a more comprehensive and effective peer review. Providing a brief summary of what the systematic review has reported is an important first step in the peer review process (and not performed frequently enough). At its core, it provides the authors with some sense of what the peer reviewer believes was performed (Methods) and found (Results). Importantly, it also provides clarity regarding any potential problems in the methods, including statistical approaches for meta-analysis, results, and interpretation of the systematic review, for which the peer reviewer can seek explanations from the authors; these clarifications are best presented as questions to the authors.

## Introduction

Two days ago you received an electronic correspondence from an editor of a journal inviting you to peer review a systematic review and meta-analysis (henceforth called systematic review). The editor asked you to let the journal know whether you will agree to the invitation or decline it in a couple of days. The request for the quick response is typically an effort by the journal to keep the peer review process to as short a time period as possible. Having reviewed the abstract, and author list, to ensure no obvious conflict(s) of interest, you have agreed to the peer review request.

If you are an experienced peer reviewer you will be familiar with the process required to successfully complete your review. However, if you have limited experience peer reviewing there are some excellent resources, such as those discussed in Patel’s recent blog series on peer review [1–3]. Additionally, reviewers may wish to consider discussing the process with a more senior colleague and/or mentor; however, if help is received to complete the review, it is important that editors are informed about these details.

## What to consider when invited to peer review a systematic review

Peer reviewers should initially assess whether the journal has approached them for an opinion as a content expert, methodologist, or both, or perhaps this was not indicated in the editor’s request. If the reviewer’s area of expertise is methodology, it is probably best to minimize the time spent commenting on the content area of the systematic review. Content experts are often formally trained in methodology through, for example, a Masters degree in clinical epidemiology, and can provide excellent content and methodological feedback.

Reports of systematic reviews are typically longer and include more tables, figures, and appendices than other types of research articles. A general plea to authors submitting systematic reviews for consideration of publication is the use of continuous line numbering in the manuscript to facilitate easier peer reviewing; it allows the reviewer to specify the exact line(s) in the manuscript to which comments refer to and saves the reviewer time counting the lines in the manuscript.

A further immediate point to assess is whether the journal has provided a template to complete the peer review report. Even if this is the case, supplementing this with a reporting guideline checklist (see below for information about the EQUATOR Network) is highly recommended. Examining the checklist items provides a memory jog for the reviewer to assess the degree to which the authors have reported on each of these and to seek clarification where there is ambiguity, incomplete reporting, or regarding other pre/clinical content and/or methodological concerns. Many journals, including *BMC Medicine*, recommend using reporting guidelines as part of the peer review process. There is emerging evidence to support this guidance. Cobo et al. [4] have shown that the use of a reporting guideline during the peer review process improves the quality of the final publication. A comprehensive list of reporting guidelines can be found on the EQUATOR Network’s library of reporting guidelines [5]. Patel also discusses EQUATOR in her second blog on peer review [2]. For systematic reviews, several reporting guideline options are available (Table 1).

**Table 1:** Examples of reporting guidelines available for peer reviewers assessing systematic reviews

Type of systematic review	Helpful reporting guideline for peer reviewing
Systematic reviews of randomized controlled trials evaluating healthcare interventions	The Preferred Reporting Items for Systematic reviews and Meta-Analysis (PRISMA) [22]; hundreds of biomedical journals endorse PRISMA
Systematic reviews of observational studies	Meta-analysis of observational studies in epidemiology (MOOSE) [23]
Systematic reviews involving psychological interventions	The Meta-Analysis Reporting Standards (MARS) guidance [24]; The American Psychological Association endorses MARS.
Synthesis of qualitative studies	The ENhancing Transparency in REporting the synthesis of Qualitative research (ENTREQ) [25]
Realist reviews	Realist And Meta-narrative Evidence Synthesis – Evolving Standards (RAMESES) for reviewing meta-narrative reviews and realist reviews [26, 27]

## A tip for peer reviewers

The checklist items covered by PRISMA are relevant to any systematic review, not just those summarizing the benefits and harms of a healthcare intervention, although some adjustments should be considered. For example, assessing the risk of bias is a key concept, but the items used to assess this in a diagnostic review are likely to focus on issues such as the spectrum of patients and the verification of disease status, which differ from reviews of interventions.

The PRISMA Statement includes a lengthy explanation and elaboration document explaining to authors why specific information should be included in the report of a systematic review [6]. This paper can also be helpful for peer reviewers. However, since its publication in 2009, the PRISMA Statement has not yet been updated and peer reviewers should be aware of the new and emerging literature regarding methods and specific content, for both clinical and pre-clinical research. Such knowledge adds to the breadth and depth of a peer review. For example, in 2010, Sampson et al. [7] developed the Peer Review of Electronic Search Strategies (PRESS) to help reduce errors found in published search strategies, a fundamental step in the systematic review process. *Another tip for peer reviewers*: it is important to assess whether the authors have indicated if their search strategy underwent any type of peer review and seek clarification if this information cannot be found in the manuscript.

A general suggestion is to keep the peer review report to one, single spaced, double-sided page, which equates to approximately 1000 words, sufficient to provide a thoughtful, meaningful, and concise review. There is little evidence as to whether lengthy peer review is more useful than shorter, more concise reports. Another strong suggestion is to keep the peer review as evidence-based as possible [8]; As a peer reviewer it is important to be as complete and transparent in the feedback to the authors, ideally supporting comments with the best available evidence, whenever possible. For example, if the authors of a systematic review do not appear to have assessed for the risk of bias in the included studies, this is problematic as there is strong evidence that inadequate reporting of randomization can exaggerate the estimates of treatment effect [9, 10]. As a peer reviewer, it is appropriate to ask the authors for clarification as to why they did not assess for risk of bias, providing them with an explanation and including references as to why this is important in the conduct of a systematic review. A final general recommendation is keep personal opinion about the systematic review to a minimum, and to never be rude in any comments to the authors.

## Completing the peer review

A peer review has good face validity when it starts with a summary of the systematic review [2]. This can be achieved in a short paragraph summarizing what the authors performed (Methods) and found (Results). The précis gives the authors (and editor) a sense that the reviewer has correctly summarized the most salient aspects of the systematic review. Following this, it is often helpful to continue the peer review report mentioning positive aspects of the manuscript. For example, perhaps the authors have addressed a very topical issue, such as the health effects of sugar-sweetened beverages, or the eligibility criteria for studies considered for the review is well thought through and documented. There is little benefit in being negative about everything in the manuscript. In general, authors make a substantial effort to report their review and may have spent considerable time on their manuscript preparation.

The next part of the peer review report should focus on any major problem(s) detected, including fatal flaw(s). For example, the authors may have not reported on the completion of an electronic search for potentially eligible articles and instead relied on studies contained in their personal files, or they may have not reported on the performance of any risk of bias assessments of the included studies. It is often useful to pose these problems as questions for clarification to the authors, since the authors perhaps simply forgot to report them. Alternatively, they may not have considered the preclinical or clinical issues or methods enquired about by the reviewer. There is considerable evidence that the completeness for reporting of most published research, including systematic reviews, is not optimal [11]. Effective peer reviewers need to be as familiar as possible with recent advances in the literature related to their area of expertise. For example, perhaps the study is examining the effectiveness of yoga versus pharmacotherapy for the management of mild to moderate depression in teenagers. As a content expert in depression, the reviewer may be aware of two other recently published reviews addressing the same question, yet the authors have not cited these studies or provided a rationale as to why their study was undertaken; an effective peer reviewer should seek clarification from the authors on these points.

Selective reporting of outcomes is a serious and prevalent problem in clinical and pre-clinical research, including systematic reviews [12–14]. The optimal method for peer reviewers to examine for outcome reporting bias is to compare the outcomes reported in the completed review against those documented in the protocol. Peer reviewers can do this in different ways. If the report included a PROSPERO [15] registration number, it is possible to examine the registration entry against the completed report. Alternatively, an increasing number of systematic review protocols are being published in journals, such as *Systematic Reviews* and *BMJ Open*, and referenced in the completed review. Examining the published protocol against the completed review provides another opportunity to assess for potential reporting biases.

Approximately half of systematic reviews include at least one meta-analysis. Some of these analyses have statistical issues which could have likely been detected during peer review. These systematic reviews require special attention and should have a separate section in the peer review report. For example, have the authors indicated the statistical approach used? Do they provide confidence intervals along with reporting of any point estimates? Have they assessed for the presence of publication bias, and, if so, have they reported using a graphical method only (funnel plot) and a statistical test (e.g. Egger test)?

Reports of systematic reviews can present with several inherent issues. Often, peer reviewers may request numerous substantial modifications to the systematic review, such as modification of the eligibility criteria to include studies with a different dose of pharmacotherapy or an additional study design. It is not always useful asking authors to what amounts to conducting a new systematic review. Peer reviewers should remember that they have been asked to peer review a completed systematic review. Indeed, such issues might be marker for recommending rejection (confidentially to the editor).

Once comments regarding any major concerns with the systematic review have been presented, other relevant but less serious issues should be indicated. For example, the review authors have indicated the completion of a meta-analysis although there is no indication as to whether they used a fixed-effects, random-effects, or Bayesian approach. Seeking clarification regarding this point would help interested readers replicate the analytical methods used by the authors.

Sometimes the discussion section of the systematic review goes beyond the results or the results are interpreted too optimistically; this is termed spin and has been noted in systematic reviews [16]. An effective peer reviewer will assess the discussion section of the review with particular reference to any possibility of spin, seeking clarification from the authors whenever it is suspected. Peer reviewers should also assess the discussion section to see whether the authors have commented on limitations of the review itself, including those of the included studies.

Apart from traditional systematic reviews there are now many other types of reviews, such as rapid reviews, scoping reviews, individual participant data meta-analyses, and network meta-analyses [17]. Some of these reviews often require a deeper knowledge and understanding of statistics. Similarly, an increasing number of systematic reviews include mixed methods approaches. Peer reviewers should not feel obliged to take on the task of peer reviewing these reports unless they are familiar with the methods. The PRISMA Statement has been extended to include some of these designs [18, 19] and includes relevant checklists that can facilitate the peer review process.

Finally, when invited to peer review a protocol of a systematic review, reviewers might find the PRISMA-P checklist helpful [20, 21]. Information and access to all of these checklists can be found at the EQUATOR Network’s comprehensive library of reporting guidelines [5].

## Conclusions

Systematic reviews come in all types of shapes and sizes; there are several reporting guidelines that can facilitate the peer review process. Peer reviewers should ensure that they provide the authors with a brief summary of their report, followed by a review of any fatal/major flaws detected or any other concerns; posing these concerns as questions and clarifications to the authors is far better than being accusatory or rude. Peer reviewers play an important role in helping ensure that published systematic reviews are of the highest possible quality. In practical terms, this means that the systematic review should be completely and transparently reported and that the methods can be reproduced by interested readers.
